# Sensitive Aflatoxin B1 Detection Using Nanoparticle-Based Competitive Magnetic Immunodetection

**DOI:** 10.3390/toxins12050337

**Published:** 2020-05-20

**Authors:** Jan Pietschmann, Holger Spiegel, Hans-Joachim Krause, Stefan Schillberg, Florian Schröper

**Affiliations:** 1 Fraunhofer Institute for Molecular Biology and Applied Ecology IME, Forckenbeckstraße 6, 52074 Aachen, Germany; jan.pietschmann@ime.fraunhofer.de (J.P.); holger.spiegel@ime.fraunhofer.de (H.S.); stefan.schillberg@ime.fraunhofer.de (S.S.); 2 Institute of Biological Information Processing, Bioelectronics IBI-3, Forschungszentrum Jülich, 52428 Jülich, Germany; h.-j.krause@fz-juelich.de

**Keywords:** frequency mixing technology, immunofiltration, magnetic beads, mycotoxin

## Abstract

Food and crop contaminations with mycotoxins are a severe health risk for consumers and cause high economic losses worldwide. Currently, different chromatographic- and immuno-based methods are used to detect mycotoxins within different sample matrices. There is a need for novel, highly sensitive detection technologies that avoid time-consuming procedures and expensive laboratory equipment but still provide sufficient sensitivity to achieve the mandated detection limit for mycotoxin content. Here we describe a novel, highly sensitive, and portable aflatoxin B1 detection approach using competitive magnetic immunodetection (cMID). As a reference method, a competitive ELISA optimized by checkerboard titration was established. For the novel cMID procedure, immunofiltration columns, coated with aflatoxin B1-BSA conjugate were used for competitive enrichment of biotinylated aflatoxin B1-specific antibodies. Subsequently, magnetic particles functionalized with streptavidin can be applied to magnetically label retained antibodies. By means of frequency mixing technology, particles were detected and quantified corresponding to the aflatoxin content in the sample. After the optimization of assay conditions, we successfully demonstrated the new competitive magnetic detection approach with a comparable detection limit of 1.1 ng aflatoxin B1 per mL sample to the cELISA reference method. Our results indicate that the cMID is a promising method reducing the risks of processing contaminated commodities.

## 1. Introduction

According to the Food and Agriculture Organization of the United Nations and a recent study by Eskola et al. in 2019, approximately 25% of food-crops worldwide are contaminated with mycotoxins, a group of secondary metabolites produced by molds [1,2]. Particularly, the toxins of *Aspergillus, Fusarium,* and *Penicillium* species are the most detrimental ones because these so-called aflatoxins, ochratoxins, trichothecenes (especially deoxynivalenol), and zearalenone have various adverse effects on the health of humans and animals [3,4,5,6,7,8]. From all mycotoxins, aflatoxin B1 (AFB1), mainly produced by *Aspergillus* species, has the strongest adverse effects on health, as it might lead to liver cancer [9,10,11,12]. Several factors can promote the enrichment of mycotoxins within food products. Key drivers are late harvests of crops as well as elevated humidity and temperature during storage. Within the EU, but also in the USA, Brazil, and other countries, regulatory requirements regarding the maximum tolerable levels of mycotoxins in various foods have been established. Regulatory limits in the EU for the IARC group 1 carcinogen aflatoxin B1 are set to 2–8 ng/g (ppb; ng·mL^−1^) in grain, corn, nuts, and fruits, as published in (EG) Nr. 1881/2006. Especially due to those harsh effects and corresponding low regulatory limits, highly sensitive and reliable detection technologies are of primary importance.

Currently, there are basically three analytical technologies used for mycotoxin testing. On the one hand, laboratory-based liquid chromatography (LC) coupled to mass spectrometry (MS) is the most common method, as well as enzyme-linked immunosorbent assay (ELISA), are routinely used for highly sensitive mycotoxin testing. On the other hand, lateral flow assays (LFA) are used for fast but less sensitive on-field testing [13,14,15,16,17,18,19]. Although LC-MS/MS methods have the great advantage of high sensitivity and simultaneous detection of currently more than 500 mycotoxins within a single run, expensive equipment, highly qualified staff, and a possible complex sample cleanup inhibit the applicability for fast on-site testing [20]. In comparison to LC-based methods, ELISA techniques are cheaper and, according to Renauld and colleagues (2019), faster in assay procedure, but also require a lab with the corresponding equipment for sample preparation and analysis [21].

In the field of ELISA, the most commonly used format for aflatoxin detection is the competitive assay because of the small molecular structure of the antigen that prevents the simultaneous binding of two antibodies, which would be the prerequisite for a sandwich ELISA. The basic working principle is the coating of mycotoxin-conjugate onto a microtiter plate followed by the addition and incubation of a mycotoxin-specific antibody in the presence of a liquid extract of sample material. In this step, mycotoxins in the sample compete with coated mycotoxins for antibody-binding. Subsequently, antibodies saturated with soluble mycotoxins are removed during a washing step and cannot contribute to a signal, achieved by either direct readout or indirect readout by means of a labeled secondary antibody. In this format, a low signal corresponds to a high concentration of mycotoxin within the sample, and vice versa. Most of the currently commercially available ELISA-based assay formats have a limit of detection (LOD) ranging from 1 µg·mL^−1^ (equals ng·mL^−1^ or ppb) up to 50 µg·mL^−1^, for example, Ridascreen Aflatoxin B1 30/15 test kit (r-biopharm, Darmstadt, Germany) or AgraQuant Aflatoxin B1 ELISA test (Romer Labs, Butzbach, Germany). Nevertheless, due to the requirement for laboratory equipment, their usability for on-site testing is very limited. In such cases, commercially available LFA have the highest potential for fast and cost-effective on-site mycotoxin testing. However, most of these assay systems provide only qualitative results with LODs ranging from 4 ppb to 5 ppb, as achieved with RIDA^®^Quick Aflatoxin test kit(r-biopharm, Darmstadt, Germany) or AFB1 (Aflatoxin B1) lateral flow assay kit (Elabscience, Texas, USA), respectively. Primarily due to matrix interference effects, these assays can result in false-positive detection [22,23].

**Figure 1:**
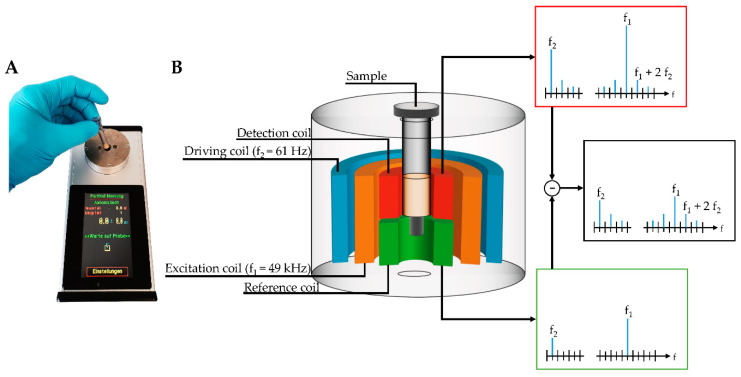
(**A**) Handheld, portable frequency mixing-based readout device and (**B**) schematic cross-section of detection head composed of driving coil providing the low driving frequency (f_2_), the excitation coil providing the high excitation frequency (f_1_) and detection unit based on a detection coil detecting the resulting mixing frequency signal of magnetic particles (MPs; f_1_ + 2f_2_) together with the directly induced signal and the reference coil detecting only the directly induced signal. The finally resulting measuring signal does not contain the directly induced excitation due to the opposite winding direction of detection and reference coil.

A novel, portable magnetic immunodetection approach has been described in previous studies [24,25,26,27,28]. The detection of human and plant pathogens, as well as various proteins by sensing and quantifying superparamagnetic particles (MP) with the help of a portable magnetic reader (Figure 1A), have been successfully demonstrated. This device can be operated using a conventional external power adapter or a portable battery, allowing an on-site readout without electrical infrastructure. For this immunomagnetic detection approach, MPs were functionalized with monoclonal antibodies directed against target molecules, retained in a sandwich-based manner within an immunofiltration column, and can be detected by means of frequency mixing magnetic detection (FMMD) technology [29].

In this technique, magnetic particles are subjected to two sinusoidal magnetic fields of different frequencies generated by two excitation coils, which are schematically shown within the measurement head in Figure 1B. Here, MPs are exposed to a low- and high-frequency magnetic field, so-called driving frequency, generated by the outermost coil, and excitation frequency, generated by the middle-positioned coil. The low frequency with 61 Hz (f_2_) has an amplitude of a few millitesla, resulting in alternating positive and negative magnetic saturation of superparamagnetic particles oscillating with a frequency of 2f_2_ of 122 Hz [29]. The high-frequency magnetic excitation field (f_1_) with 49 kHz probes the magnetization state of the superparamagnetic particles and yields an iron oxide dose-dependent signal when the low-frequency driving field is close to zero. Finally, the resulting mixing frequency signal of f_1_ + 2f_2_ can be demodulated and detected by the innermost coil, composed of two adjacent sections, so-called detection coil (upper one) and reference coil (lower one). Those sections differ only in the winding-orientation of coils. With this clock- and counterclockwise orientation yielding induced voltages of opposite sign, the directly induced excitation field can be canceled out. By placing the sample carrying the MPs in the detection head, the resulting signal is amplified, measured, and directly visualized at the touchscreen of the handheld, portable magnetic reader (Figure 1). Based on a calibration curve, the detected signal can be attributed to the amount of analyte within the sample.

**Figure 2:**
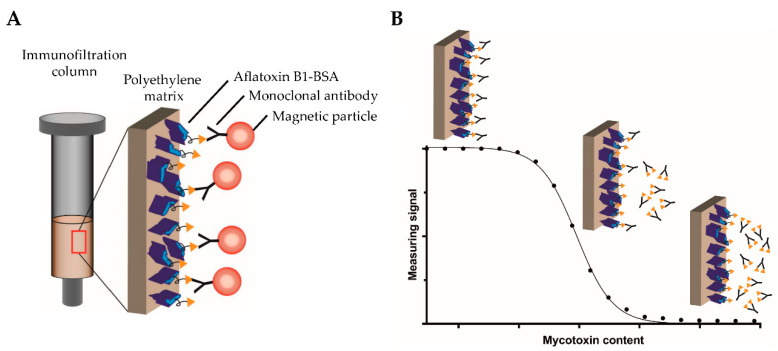
Schematic overview of the competitive magnetic immunodetection principle. (**A**) Immunofiltration column coated with aflatoxin B1-BSA mycotoxin conjugate with bound biotinylated monoclonal antibodies targeting aflatoxin B1. Magnetic particles functionalized with streptavidin bind to antibodies and can be detected by FMMD. (**B**) After pre-incubation of biotinylated, monoclonal antibodies with serially diluted free aflatoxin B1, the sample is flushed through an aflatoxin B1-BSA coated immunofiltration column. Non-saturated antibodies bind to the coated antigen and are retained within the matrix. The higher the mycotoxin content within the sample, the more antibodies are saturated and are flushed through the column. Afterward, streptavidin-functionalized magnetic particles are applied onto the column, bind to retained antibodies and can be detected using FMMD.

Motivated by the above-described drawbacks of currently used analytical methods for sensitive on-field testing, the aim of this study was to develop a novel, highly sensitive, and portable assay based on competitive magnetic immunodetection (cMID) and FMMD. The sensitivity should be comparable to a laboratory-based ELISA. Hence, initially, a competitive ELISA (cELISA) was established, serving as a reference method. Assay parameters, such as the used coating and antibody concentrations, were optimized to reach a sufficient sensitivity for the detection of aflatoxin B1. Afterward, the cMID assay was established using the same optimization strategy in combination with further evaluation of the required amount of nanoparticles. The basic principle of cMID is the use of biotinylated antibodies, which can be enriched within the coated immunofiltration column by a competitive binding reaction depending on the amount of pre-captured mycotoxin. By flushing magnetic particles functionalized with streptavidin through the column by gravity flow, particles can bind to retained antibodies and subsequently be detected using FMMD. Figure 2 visualizes the basic cMID principle (Figure 2A) and the competitive binding reaction within the column with the corresponding measuring signal (Figure 2B).

## 2. Results

### 2.1. Competitive ELISA Conditions

As a reference method to novel cMID, a cELISA was established using optimized conditions defined by checkerboard titration. Here, aflatoxin B1-BSA conjugate was coated, followed by the application of aflatoxin B1-specific monoclonal antibodies pre-incubated with soluble aflatoxin B1 with concentrations ranging from 0.006 ng·mL^−1^ to 5000 ng·mL^−1^ sample buffer. Absorbance was measured at 405 nm by indirect readout using a secondary antibody conjugated to horseradish peroxidase (HRPO) targeting mouse antibodies and application of respective substrate.

#### 2.1.1. ELISA Checkerboard Titration Test

**Figure A1:**
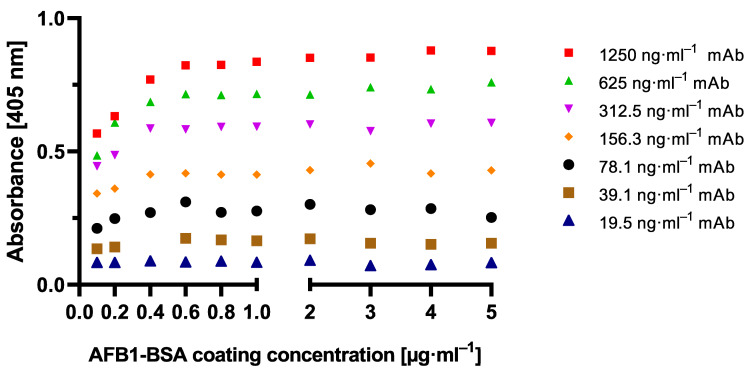
ELISA checkerboard titration determining suitable aflatoxin B1-BSA (AFB1-BSA) coating concentrations ranging from 0.1 µg·mL^−1^ to 5 µg·mL^−1^ and monoclonal antibody (mAb) AFB1_002 concentrations ranging from 19.5 ng·mL^−1^ to 1250 ng·mL^−1^. Absorbance was measured at 405 nm by indirect readout after using a mouse-specific secondary antibody conjugated to horseradish peroxidase and application of respective substrate (*n* = 1).

In order to determine optimal aflatoxin B1-BSA (AFB1-BSA) coating concentrations and the appropriate amount of aflatoxin B1-specific monoclonal antibody to obtain the highest possible sensitivity, a checkerboard titration test was performed. As shown in Figure A1, AFB1-BSA coating concentrations ranging from 0.1 µg·mL^−1^ up to 5 µg·mL^−1^ in combination with aflatoxin B1-specific monoclonal antibody (mAb) AFB1_002 concentrations ranging from 19.5 ng·mL^−1^ up to 1250 ng·mL^−1^ were tested in a 96-well plate. Increasing signals were achieved with antibody concentrations above 19.5 ng·mL^−1^ and coating concentrations up to 0.6 µg·mL^−1^. Coating of AFB1-BSA with higher amounts than 0.6 µg·mL^−1^ resulted in saturated readout signals at all antibody concentrations that did not further increase. Considering sensitivity, the lowest possible amount of antibody should be used with coating concentrations resulting in highest possible signals. As a consequence, combinations of 75 ng·mL^−1^ or 150 ng·mL^−1^ antibody with 0.2 µg·mL^−1^ and 0.4 µg·mL^−1^ coating of AFB1, respectively, seem to be best suited for further experiments.

#### 2.1.2. cELISA Calibration Curve Experiments

**Figure 3:**
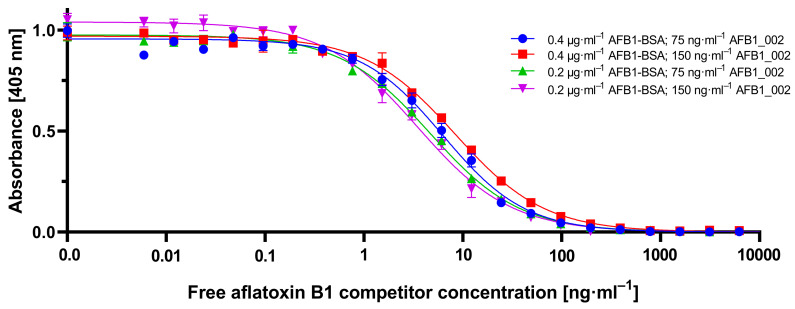
Competitive ELISA-based calibration curves of different pairs of aflatoxin B1-BSA coating and aflatoxin B1-specific monoclonal antibody AFB1_002 concentrations pairs. As a competitor, free aflatoxin B1 in dilutions ranging from 0.006 ng·mL^−1^ up to 50,000 ng·mL^−1^ in sample buffer was used. The indirect readout was done at 405 nm after the application of mouse-specific secondary antibody conjugated with horseradish peroxidase and respective substrate. Each data point represents the mean ± SD (*n* = 3).

To identify the optimal assay parameters, calibration experiments were performed with the previously most promising combinations of AFB1-BSA coating and aflatoxin B1-specific monoclonal antibody AFB1_002 concentrations (Figure 3). Free aflatoxin B1 was used as a competitor, dilutions ranging from 0.006 ng·mL^−1^ to 50,000 ng·mL^−1^. The combination of 0.2 µg·mL^−1^ coating and 150 ng·mL^−1^ antibody resulted in the highest sensitivity in combination with stable data behavior. Corresponding IC_50_ and Limit of Detection (LOD) values of all four combinatorial experiments are shown in Table 1. Although antibody concentrations below 75 ng·mL^−1^ should theoretically lead to a higher sensitivity, this was actually not observed due to divergent measuring values and thus a non-reliable assay procedure under these conditions (compare 0.4 µg·mL^−1^ coating and 75 ng·mL^−1^ mAb). Furthermore, reducing the amount of mAb resulted in a twofold increase of readout time from approximately 10 min to more than 20 min (data not shown).

**Figure 4:**
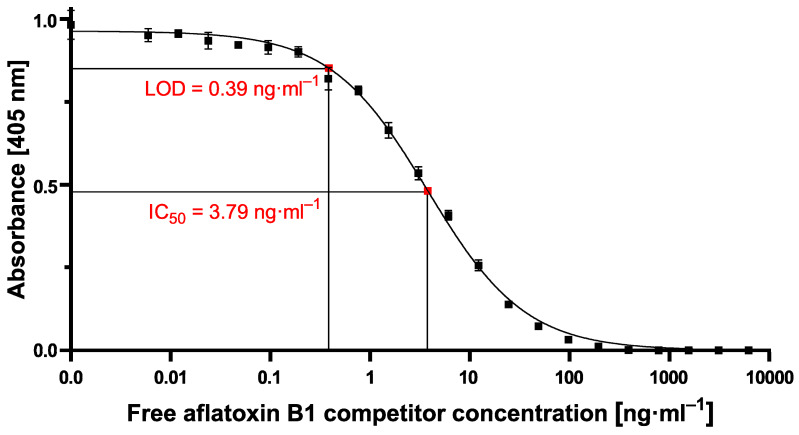
Averaged calibration curve with 0.2 µg·mL^−1^ AFB1-BSA conjugate coating and 150 ng·mL^−1^ mAb with aflatoxin B1 competitor concentrations ranging from 0.006 ng·mL^−1^ to 500,000 ng·mL^−1^. Measuring signal was achieved by indirect readout at 405 nm with mouse-specific secondary antibody conjugated to horseradish peroxidase and respective substrate LOD: limit of detection; IC_50_: half maximal inhibitory concentration. Each data point represents the mean ± SD (*n* = 12).

Considering the reliability of the developed cELISA, a further repetition of the best-paired concentration setting, as shown in Table 1, was done. Results yielded in an averaged IC_50_ value of 3.79 ng·mL^−1^ and an averaged LOD of 0.39 ng·mL^−1^, as shown in Figure 4. Those sensitivity values obtained by our ELISA setup were used as a reference for competitive magnetic immunodetection development.

### 2.2. Competitive Magnetic Immunodetection

#### 2.2.1. Development of Competitive Magnetic Immunodetection

**Figure A2:**
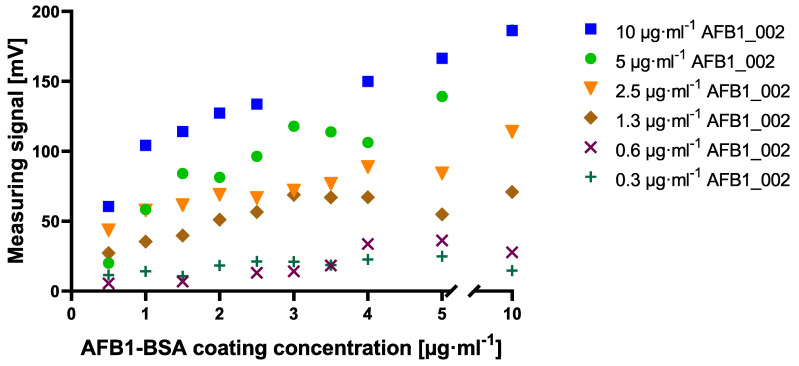
Magnetic immunodetection checkerboard titration determining suitable aflatoxin B1-BSA (AFB1-BSA) coating concentrations and aflatoxin B1-specific biotinylated monoclonal antibody AFB1_002 concentrations. The readout was done by detecting 700 nm streptavidin-functionalized magnetic particles coupled to biotinylated AFB1_002 using FMMD (*n* = 1).

In analogy to the development of the cELISA, a similar strategy was used for establishing suitable cMID conditions regarding AFB1-BSA coating and suitable antibody concentrations. For this purpose, equilibrated polyethylene filters were coated with AFB1-BSA conjugate concentrations ranging from 0.5 µg·mL^−1^ up to 10 µg·mL^−1^ with one column per condition. Remaining binding sites were blocked with a BSA solution. Then aflatoxin B1-specific biotinylated monoclonal antibody AFB1_002 in concentrations ranging from 0.3 µg·mL^−1^ up to 10 µg·mL^−1^ were applied onto these columns after pre-incubation with 180 µg·mL^−1^ of 700 nm superparamagnetic streptavidin-functionalized particles (Figure A2). After rinsing the columns with PBS by gravity flow, the columns were inserted into the handheld magnetic readout device and superparamagnetic particles were excited by frequency mixing technology resulting in a response signal in the millivolt (mV) range after amplification.

Especially in the range of low antibody concentrations between 0.3 µg·mL^−1^ and up to 1.3 µg·mL^−1^, a saturation of measurement signal was reached at higher coating concentrations. In contrast, using higher antibody concentrations, no clear saturation of signal was observed even at higher coating concentrations. Furthermore, using antibody concentrations of 5 µg·mL^−1^ or 10 µg·mL^−1^ resulted in high and erratic signal variability. As explained in Section 2.1.1, the lowest possible antibody concentration should be used for obtaining the highest possible sensitivity. On the other hand, reducing the antibody concentrations leads to a reduction of possible binding sites for magnetic particles and, as a consequence, to a reduction in measuring signal, which may limit the dynamic range as well as the overall robustness of the assay. To obtain the highest possible measuring signal in combination with the highest possible sensitivity, 2 µg·mL^−1^ coating in combination with 2.5 µg·mL^−1^ biotinylated antibody were used.

**Figure 5:**
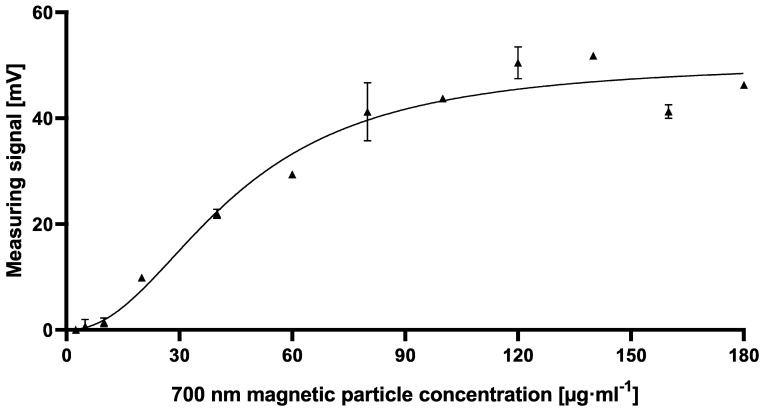
Dose-dependent measuring signal of 700 nm streptavidin-functionalized magnetic particles after applying 2.5 µg·mL^−1^ biotinylated AFB1_002 monoclonal antibody onto 2 µg·mL^−1^ AFB1-BSA coated immunofiltration columns. Each data point represents mean ± SD (*n* = 2).

As shown in Figure A2, the measuring signal increased with higher antibody concentrations. Therefore, it can be assumed that at the used concentrations of 180 µg·mL^−1^ there is still an excess of magnetic particles when using 2.5 µg·mL^−1^ biotinylated mAb. To test this hypothesis, different amounts of 700 nm magnetic particles ranging from 2.5 µg·mL^−1^ to 180 µg·mL^−1^ were flushed through immunofiltration columns coated with 2 µg·mL^−1^ AFB1-BSA conjugate after applying 2.5 µg·mL^−1^ biotinylated antibody to the matrix (Figure 5). The expected saturation of measuring signal was observed when using more than 80 µg·mL^−1^ magnetic particles. Adding 80 µg·mL^−1^ resulted in 41.2 mV ± 5.5 mV, in comparison to higher bead concentrations with averaged signals of 47.4 mV ± 4.1 mV. For further experiments, 80 µg·mL^−1^ magnetic particle suspension was used.

#### 2.2.2. cMID Calibration Curve Experiments

**Figure 6:**
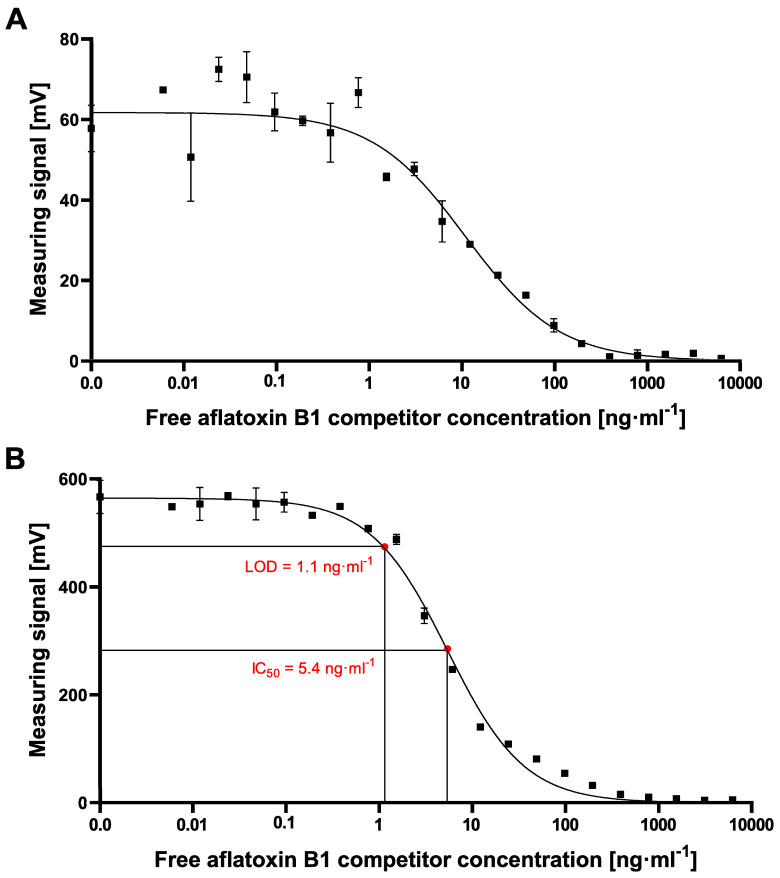
cMID calibration curves with 2 µg·mL^−1^ AFB1-BSA conjugate coating and 2.5 µg·mL^−1^ biotinylated mAb in combination with (**A**) 700 nm streptavidin-functionalized magnetic particles, and (**B**) 70 nm streptavidin-functionalized magnetic particles with aflatoxin B1 competitor concentrations ranging from 0.006 ng·mL^−1^ to 500,000 ng·mL^−1^. LOD, limit of detection; IC_50_, half maximal inhibitory concentration. Each data point represents the mean ± SD (*n* = 2).

Once the optimal ratio of assay reagents of 2 µg·mL^−1^ of the coating antigen and 2.5 µg·mL^−1^ of mycotoxin-specific antibody in combination with 80 µg·mL^−1^ magnetic particles was determined based on the above-shown experiments, cMID calibration experiments for the detection of aflatoxin B1 were performed, see Figure 6A. For this, samples with serially diluted free AFB1 in the range of 0.006 ng·mL^−1^ to 50,000 ng·mL^−1^ were prepared. After adding biotinylated mAb to various dilutions of the analyte, a pre-incubation of one hour was done for a complete capturing of mycotoxins. Subsequently, the reaction mixture was applied onto AFB1-BSA coated and blocked columns. Afterward, 700 nm diameter magnetic particles functionalized with streptavidin at the above-determined optimum concentration of 80 µg·mL^−1^ were added. As shown in Figure 6A, the data show the expected reciprocal correlation of mycotoxin concentration and signal but with high variability of signals for dilutions in the low- ng·mL^−1^ range, which prevents a reliable determination of sensitivity parameters. We speculate that this observation could be caused by sterical hindrance within the polyethylene matrix due to the usage of big-sized particles, as can be seen in the standard deviation and data fluctuation with bead concentrations of 80 µg·mL^−1^ or higher (Figure 5). Based on these findings, the assay was repeated with the same parameters except the size of beads used. For this, tenfold smaller magnetic particles were used (70 nm) in order to avoid steric hindrance. As shown in Figure 6B, the use of smaller particles results in an approximately tenfold increased detection signal of roughly 600 mV with reduced variability. With this adapted assay procedure, IC_50_ values of 5.4 ng·mL^−1^ and a LOD of 1.1 ng·mL^−1^ were determined, which is in the same sensitivity range as the cELISA (Figure 4).

## 3. Discussion

In this study, we demonstrated for the first time, a competitive magnetic immunodetection assay for efficient detection and quantification of aflatoxin B1 with comparable sensitivity to described laboratory-based cELISA. Especially due to the handheld reader device which can be operated using a portable battery and without additional laboratory equipment, the method described here is suitable for sensitive on-site aflatoxin B1 testing. Commonly used analytical methods for detection of mycotoxins either exhibit high sensitivity in a laboratory-based setting or can be used on-site with reduced sensitivity [21].

To establish the novel cMID method, several concentration settings of a cELISA as reference method were tested and optimized using checkerboard titration (Figure A1). Here, the most suitable combination of antigen coating concentration and the amount of used antibody was determined. As explained in Section 2.1.1, in all tested antibody concentrations, for a coating of 0.4 µg·mL^−1^ and onwards, a saturation in measuring signal could be detected (Figure A1). A clear difference to this can be seen within the checkerboard optimization experiment for cMID (Figure A2). Here, a saturation-like performance can either be seen when using antibody concentrations up to 2.5 µg·mL^−1^ or, if further increasing antibody concentrations, saturation cannot be reached. This could be due to the more than 40-fold higher protein binding surface of immunofiltration columns in comparison to the binding surface of an ELISA microtiter plate well. Especially due to the highly porous membrane of the immunofiltration column, a higher protein binding capacity can be reached. High binding 96-well microtiter plates (Greiner Bio-One, article number 655061) have a maximum binding capacity of 600 ng·cm^2^, whereas ABICAP immunofiltration columns can bind up to 24,000 ng·cm^2^, which in fact explains the absence of signal saturation at the used conditions. A further difference when using checkerboard titration for assay optimization of cELISA or cMID can be seen in fluctuating readout signals, whereas in ELISA, concentration-dependent signals were obtained with almost no variability between consecutive measuring points. In contrast, signals generated in MID checkerboard titration varied, especially at high antibody concentrations. This could be caused by an overdose of magnetic particles, which might result in sterical hindrances and a partly erratic aggregation of particles within the assay matrix. Based on this hypothesis, a magnetic-particle dose adaptation was made by reducing the applied concentration towards robustly detectable saturated measuring signals (Figure 5).

However, the resulting cMID assay did not lead to the expected results when using 700 nm magnetic beads. Switching to 70 nm magnetic particles instead resulted in a tenfold increase of measuring signal combined with reduced variability and provides a basis for a highly sensitive and reliable assay (Figure 6). The results presented here correlate with data published by Achtsnicht et al. in 2019, where the authors used magnetic frequency mixing for the detection of cholera toxin subunit B (CTB) in a sandwich-based manner [27]. In their experiments, using magnetic particles with hydrodynamic diameters of 75 nm and 1010 nm, they observed an eight-fold higher response signal with smaller particles compared to the larger beads when detecting a concentration of 750 ng·mL^−1^ CTB. The most simple explanation for this striking difference would be the higher absolute number of small particles compared to larger particles when used at identical concentrations (80 µg·mL^−1^) since the molar concentration is proportional to the molecular weight of the particles. By applying a higher ratio of small particles onto the column, a higher amount of small MPs could bind to antigens coated on the column surface. Another explanation could be the occurrence of steric hindrance within the ABICAP immunofiltration matrix when using large particles. Especially if there is a low concentration of free mycotoxin within the sample, a high amount of biotinylated antibody will be retained within the matrix in a competitive assay approach, resulting in a high antibody density on the matrix surface. When large MPs are applied, steric hindrance can occur at the surface. Either multiple, closely located biotinylated antibodies could be linked to one MP, or multiple antibodies could be blocked due to the size of large MPs. Here, especially the thousand-fold increased volume of 700 nm diameter MPs in comparison to 70 nm MPs in diameter might have the most important role. By the greatly increased size and volume, several antibodies are covered by one single MP, so that they are no longer accessible for binding of further MPs. By using smaller particles, shielding effects and sterical hindrances at the surface are reduced, resulting in a higher magnetic particle density within the column, and finally, a higher readout signal. A similar effect observed by Achtsnicht et al. (2019) when using small 75 nm instead of large 1010 nm particles in their MID experiments was also explained by the blocking of remaining binding sites [27]. The authors also found similar high standard deviations when large particles remain in a high concentration within the column (Figure 6A). With such a high SD, sensitive detection of either CTB or aflatoxin B1 is almost impossible. In their study, a LOD of 3.1 ng·mL^−1^ was found when using the large particles, but a more than 15-fold lower LOD of 0.2 ng·mL^−1^ could be reached using the small-sized MPs. In our study, especially due to the high standard deviations at high measuring signals when exciting the 700 nm MPs for aflatoxin B1 detection, a reliable calculation of LOD or IC_50_ values was not possible. However, by using 70 nm particles, high and stable readout signals in combination with a perfectly matching nonlinear-fit (R^2^ = 0.9938) enabled the reliable calculation of LOD and IC_50_ values of 1.1 ng·mL^−1^ and 5.4 ng·mL^−1^, respectively, which are in the same sensitivity range as our comparative lab-based cELISA

Although the best performance of the cMID assay was achieved with 70 nm small magnetic particles, the general applicability of 700 nm large particles in cMID assays should not be completely neglected since, in contrast to small particles, larger particles can be separated in a gradient magnetic field. However, the possibility of magnetic separation and thereby enrichment of magnetic particles might play a crucial role in sample preparation where cleanup and concentrating of an analyte is required to achieve sufficient assay sensitivity, as it was used for example by Lee et al. (2013) or Xuan and colleagues (2019) [30,31]. In the case of mycotoxin detection, magnetic separation-based sample cleanup could be beneficial, especially when working with partially soluble or insoluble, or roughly homogenized, e.g., grain samples. After magnetic beads captured the mycotoxin molecules in the sample, a magnetic separation step can be applied in which magnetic particles are retained in the magnetic field, while sample debris can be discarded. Additionally, separated mycotoxin-loaded magnetic particles could be resuspended in a much smaller volume, resulting in an enrichment of analyte. As a consequence, this could lead to increased sensitivity when samples are finally analyzed by cMID.

The 70 nm particles used in this study cannot be efficiently separated in a magnetic gradient field, since their magnetic attraction is low, which was shown by Achtsnicht and colleagues (2018) [32]. They correlated the magnetophoretic velocity of superparamagnetic particles when applying a magnetic field and found an increased velocity with increasing particle size [32]. Typically only bigger particles above 700 nm are used for magnetic separation experiments [30,31]. Further testing of different beads or other strategies as combining big and small-sized particles will be addressed in further studies to enable an optimized pairing of separation and cMID.

In conclusion, we successfully demonstrated the development and implementation of a competitive magnetic immunodetection assay for the detection and quantification of aflatoxin B1 with a LOD of 1.1 ng·mL^−1^. Based on our findings, it can be concluded that the competitive magnetic immunodetection is a powerful tool for portable, easy-to-use on-site monitoring of mycotoxin contamination in various matrices to reduce the risk of processing contaminated food and agricultural products. In future work, we will focus on the further optimization of the cMID procedure using the 70 nm MPs. Especially, a significant reduction of the assay time from currently approximately 4.5 h to less than one hour should be addressed, similar to that described by Rettcher et al. (2015) [26]. There a sandwich-based MID assay lasting less than 30 min was achieved by a more than 50% reduction of initially needed assay time [26]. By using MPs, pre-conjugated with specific anti-mycotoxin antibodies, ready-to-use coated and blocked immunofiltration columns, and testing successive reductions of incubation times of sample-pre-incubation and competitive binding reaction within the matrix, an additional increase of applicability for on-site testing should be possible. The applicability of the described cMID approach will be further studied regarding the detection and quantification of other mycotoxins, as well as a combination of those toxins within a food matrix of one sample. Especially the multiplex detection of various mycotoxins within one sample will be addressed as shown by the multiplex detection of several antibodies in stacked sample matrices by Achtsnicht et al. (2019) [33]. Here the authors detected two different target molecules, namely antibodies, within one sample solution by stacking 3D printed immunofiltration columns coated with different capture antibodies in a sandwich-based MID approach. By adapting this procedure, multiple individually coated matrices could be used for the specific retention of corresponding anti-mycotoxin antibodies in a cMID assay, and with this, a multiplex detection in a food matrix could be obtained.

## 4. Materials and Methods

### 4.1. Material and Chemicals

Dimethyl sulfoxide, Tween-20, Aflatoxin B1, Aflatoxin B1-BSA, EZ-Link™ NHS-PEG4 Biotinylation Kit, ABTS buffer, as well as ABTS tablets were purchased from Merck KGaA, Darmstadt, Germany. NaCl, KCl, Na_2_HPO_4_ × 12 H_2_0, KH_2_PO_4_, Na_2_(CO_3_), NaHCO_3_, Milk powder, and Albumin Fraction V (biotin-free) were acquired from Carl Roth, Karlsruhe, Germany.

The used coupling buffer was prepared by dissolving 15 mM Na_2_CO_3_ and 35 mM NaHCO_3_ in MilliQ-water, and pH was set to 9.6 with glacial acetic acid. Phosphate buffered saline (PBS) was prepared by dissolving 137 mM NaCl, 2.7 mM KCl, 8.1 mM Na_2_HPO_4_ × 12 H_2_O, and 1.5 mM KH_2_PO_4_ in MilliQ-water and setting pH to 7.4 with hydrochloric acid. As a washing buffer, PBS-T was produced by adding 0.05% (v/v) Tween-20 to PBS. ELISA blocking buffer (EBP) was prepared by adding 5% (w/v) milk powder to PBS. For magnetic immunodetection experiments, blocking solution consists of 1% (w/v) albumin fraction V (biotin free) in PBS, and is called MID-BP. All other chemicals, except Tween-20, were acquired from Roth.

Immunofiltration columns (ABICAP HP columns) were purchased from Senova Gesellschaft für Biowissenschaft und Technik mbH, Weimar, Germany. High binding 96-well microtiter plates (article number 655061) were purchased from Greiner Bio-One GmbH, Frickenhausen, Germany. Anti-mycotoxin monoclonal antibody AFB1_002 was purchased from fzmb GmbH, Bad Langensalza, Germany. Secondary antibody goat anti-mouse IgG-HRPO (article number 115-035-008) was purchased from Jackson ImmunoResearch Europe Ltd. UK. 700 nm streptavidin-functionalized magnetic particles (nanomag^®^-CLD/synomag^®^-CLD; article number 05-19-502 S09718) as well as 70 nm streptavidin-functionalized magnetic particles (synomag^®^-D, article number 104-19-701) were purchased from micromod Partikeltechnologie GmbH, Rostock, Germany.

### 4.2. Optimization of ELISA

The most suitable aflatoxin B1-BSA coating concentrations for a competitive ELISA protocol in combination with appropriate anti-aflatoxin B1 monoclonal antibody concentrations were determined by checkerboard titration. Throughout the following protocol, all incubation steps were performed at room temperature for one hour in the dark. For coating varying concentrations of AFB1-BSA, the antigen was diluted in a coupling buffer, and 100 µL per well was added to a 96-well highbinding microtiter plate (Greiner Bio-One) and incubated as mentioned above. After washing each well thrice with PBS-T, all wells were blocked using 200 µL of EBP and incubated. Then, the plate was washed again, and concentrations of monoclonal antibody ranging from 19.5 ng·mL^−1^ up to 1250 ng·mL^−1^, diluted in PBS, were added. After another washing step, as described above, 100 µL of secondary antibody goat anti-mouse IgG-HRPO, diluted 1:10,000 in PBS was added to each well and incubated. Prior to readout with 100 µL of 1 mg·mL^−1^ ATBS substrate in ABTS buffer, the plate was washed again. Absorption was measured at 405 nm after 10 min incubation in the dark.

### 4.3. cELISA Procedure

For competitive ELISA procedure, AFB1-BSA conjugate was diluted in coupling buffer and plated with 100 µL per well onto a high binding 96-well microtiter plate. As mentioned in Section 4.2, all incubation steps were performed at room temperature for one hour in the dark. After the washing step with PBS-T, each well was blocked with EBP and incubated. Meanwhile, pre-incubation of free aflatoxin B1with the monoclonal anti-aflatoxin B1 antibody was prepared. For this, 75 µL of a serial dilution of aflatoxin B1 in PBS was prepared, and then 75 µL of antibody solution diluted in PBS was added and incubated. After washing the blocked assay plate three times with PBS-T, 100 µL of pre-incubated samples were transferred to each well, respectively. After incubation, the plate was washed three times with PBS-T. Subsequently, 100 µL of conjugated goat anti-mouse IgG-HRPO secondary antibody, diluted 1:10,000 in PBS was added to each well and incubated. After washing three times with PBS-T, 100 µL of 1 mg·mL^−1^ ABTS substrate in ABTS buffer was added, and absorbance was measured at 405 nm after 15 min incubation in the dark.

### 4.4. Preparation of Immunofiltration Columns

The equilibration of immunofiltration columns was done, as described by Rettcher et al. (2015) [26]. In brief, after degassing in 96% (v/v) ethanol in a desiccator at –0.8 bar pressure, columns were washed sequentially with 750 µL 50% (v/v) ethanol-water, 750 µL water and twice 750 µL coupling buffer. Afterward, for coupling of aflatoxin B1-BSA conjugate to the matrix, the conjugate was applied to the column in gravity flow diluted in 500 µL coupling buffer and incubated for one hour at room temperature in dark surrounding. For checkerboard titration, a coating concentration, as shown in Figure A2, was used. For the bead-response curve, a coating concentration of 2 µg·mL^−1^ was applied, as well as for cMID assays. Subsequent washing of the columns was performed twice with 750 µL PBS. Remaining binding sites were blocked by applying twice 750 µL of MID-BP. After the first 750 µL flushed through the column by gravity flow, an incubation time of 5 min was set. After the second time, columns were incubated for further 30 min after they were washed again twice with PBS.

After equilibration or blocking, columns can be stored in the coupling buffer or PBS, respectively, at 4 °C for at least 14 days. In this study, a maximum storage time of one day was used.

### 4.5. Biotinylation of Anti-Aflatoxin B1 Monoclonal Antibody

For biotinylation of antibodies, the EZ-Link™ NHS-PEG4 Biotinylation Kit was used as described by the manufacturer’s instruction.

### 4.6. Optimization of cMID

After coating and blocking of immunofiltration columns, 500 µL samples of biotinylated antibody, diluted in PBS to various final concentrations between 0.3 µg·mL^−1^ and 10 µg·mL^−1^, were applied and incubated for one hour at room temperature. Afterward, a washing step was performed by rinsing two times 750 µL PBS through the column. Subsequently, for checkerboard titration, 500 µL of 180 µg·mL^−1^ 700 nm magnetic beads suspension in PBS (pH 7.4) was added and flushed through by gravity flow. For bead response analysis, various concentrations of 700 nm magnetic beads were applied. Another washing step, as described above, was performed. For readout, columns were inserted into the portable magnetic reader and the measuring signal in mV was detected, as previously described in Rettcher et al. (2015) [26].

### 4.7. cMID Calibration Curve Analysis

For cMID calibration curve experiments, a pre-incubation of free mycotoxin and biotinylated antibody was performed. For this, serially diluted aflatoxin B1 samples in PBS, with concentrations ranging from 0.006 ng·mL^−1^ to 100,000 ng·mL^−1^, were mixed 1:1 with 225 µL biotinylated antibody, also diluted in PBS. After incubation of one hour at room temperature in a dark surrounding, 500 µL of each sample was applied on coated and blocked columns and also incubated as mentioned above. After washing each column twice with 750 µL PBS, 80 µg·mL^−1^ of 70 nm or 700 nm magnetic particles were applied and incubated, as mentioned before. After washing twice, the readout was done as described above.

### 4.8. Data Analysis

For competitive ELISA as well as for competitive magnetic immunodetection and data analysis, a Hill Slope fit was done with GraphPad Prism 8.0.0. The following formulas were used to determine the LOD on the signal and on the concentration scale:

(1) $${\mathrm{Signal}}_{\mathrm{Limit}\;\mathrm{of}\;\mathrm{Detection}}={\mathrm{Average}}_{\mathrm{Sample}\;\mathrm{without}\;\mathrm{Competitor}}-3{x\;\mathrm{SD}}_{\mathrm{Sample}\;\mathrm{without}\;\mathrm{Competitor}}$$

(2) $${\mathrm{Concentration}}_{\mathrm{Limit}\;\mathrm{of}\;\mathrm{Detection}}=\left(\sqrt[{\mathrm{Hill}\;\mathrm{Slope}}]{\frac{\mathrm{Maximum}\;\mathrm{Signal}-\mathrm{Lowest}\;\mathrm{Signal}}{{\mathrm{Signal}}_{\mathrm{Limit}\;\mathrm{of}\;\mathrm{Detection}}-\mathrm{Lowest}\;\mathrm{Signal}}-1}\right)\times {\;\mathrm{IC}}_{50}\;\mathrm{ng}\cdot {\mathrm{mL}}^{-1}$$

## Figures and Tables

**Table 1:** Sensitivity values of different cELISA-based calibration measurements with different pairs of aflatoxin B1-BSA coating and aflatoxin B1-specific monoclonal antibodies.

ELISA Condition	IC_50_	Limit of Detection
0.2 µg·mL^−1^ Coating	4.7 ng·mL^−1^	0.69 ng·mL^−1^
75 ng·mL^−1^ Antibody		
0.2 µg·mL^−1^ Coating	3.5 ng·mL^−1^	0.28 ng·mL^−1^
150 ng·mL^−1^ Antibody		
0.4 µg·mL^−1^ Coating	6.3 ng·mL^−1^	0.75 ng·mL^−1^
75 ng·mL^−1^ Antibody		
0.4 µg·mL^−1^ Coating	8.4 ng·mL^−1^	0.88 ng·mL^−1^
150 ng·mL^−1^ Antibody
